# Inositol 1,4,5-Trisphosphate Receptors in Hypertension

**DOI:** 10.3389/fphys.2018.01018

**Published:** 2018-07-26

**Authors:** Ali H. Eid, Ahmed F. El-Yazbi, Fouad Zouein, Abdelilah Arredouani, Allal Ouhtit, Md M. Rahman, Hatem Zayed, Gianfranco Pintus, Haissam Abou-Saleh

**Affiliations:** ^1^Department of Pharmacology and Toxicology, Faculty of Medicine, American University of Beirut, Beirut, Lebanon; ^2^Department of Biological and Environmental Sciences, College of Arts and Sciences, Qatar University, Doha, Qatar; ^3^Department of Pharmacology and Toxicology, Faculty of Pharmacy, Alexandria University, Alexandria, Egypt; ^4^Qatar Biomedical Research Institute, Hamad Bin Khalifa University, Qatar Foundation, Doha, Qatar; ^5^Department of Biomedical Sciences, College of Health Sciences, Qatar University, Doha, Qatar

**Keywords:** aging, hypertension, Ca^2+^, IP_3_R, VSMC

## Abstract

Chronic hypertension remains a major cause of global mortality and morbidity. It is a complex disease that is the clinical manifestation of multiple genetic, environmental, nutritional, hormonal, and aging-related disorders. Evidence supports a role for vascular aging in the development of hypertension involving an impairment in endothelial function together with an alteration in vascular smooth muscle cells (VSMCs) calcium homeostasis leading to increased myogenic tone. Changes in free intracellular calcium levels ([Ca^2+^]_*i*_) are mediated either by the influx of Ca^2+^ from the extracellular space or release of Ca^2+^ from intracellular stores, mainly the sarcoplasmic reticulum (SR). The influx of extracellular Ca^2+^ occurs primarily through voltage-gated Ca^2+^ channels (VGCCs), store-operated Ca^2+^ channels (SOC), and Ca^2+^ release-activated channels (CRAC), whereas SR-Ca^2+^ release occurs through inositol trisphosphate receptor (IP_3_R) and ryanodine receptors (RyRs). IP_3_R-mediated SR-Ca^2+^ release, in the form of Ca^2+^ waves, not only contributes to VSMC contraction and regulates VGCC function but is also intimately involved in structural remodeling of resistance arteries in hypertension. This involves a phenotypic switch of VSMCs as well as an alteration of cytoplasmic Ca^2+^ signaling machinery, a phenomena tightly related to the aging process. Several lines of evidence implicate changes in expression/function levels of IP_3_R isoforms in the development of hypertension, VSMC phenotypic switch, and vascular aging. The present review discusses the current knowledge of these mechanisms in an integrative approach and further suggests potential new targets for hypertension management and treatment.

## Introduction

Cardiovascular diseases (CVD) remain the leading cause of death worldwide, with hypertension being the number one cause of this high mortality (Forouzanfar et al., 2017). Nearly one-third of the yearly global mortality is due to CVD (Chen et al., 2013). At least half or more of ischemic stroke, hemorrhagic stroke, ischemic heart disease and other CVD such as cardiomyopathy, aortic aneurysms, or peripheral vascular disease are intimately attributed to elevated blood pressure (BP), or hypertension (Forouzanfar et al., 2017). This burden is on the rise, despite all therapeutic advances made in recent years, especially in elderly people (Gates et al., 2009; GBD 2013 Risk Factors Collaborators et al., 2015; Harvey et al., 2015; GBD 2015 Risk Factors Collaborators, 2016; Thijssen et al., 2016).

Hypertension is defined as a chronic and persistent elevation of systemic arterial pressure beyond normal values. Etiologically, hypertension is classified as primary and secondary. Primary hypertension, also known as essential hypertension, is the most prevalent form of high BP and constitutes around 90–95% of the cases with unknown etiology (Carretero and Oparil, 2000; Rossier et al., 2017). Secondary hypertension, on the other hand, constitutes around 5–10% of hypertensive cases and arises from known and identifiable causes such as kidney diseases, pregnancy, endocrine disorders, neurological diseases, and others (Chiong et al., 2008).

Chronic hypertension predisposes nearly 1.5 billion individuals in the world to CVD, including ventricular hypertrophy and heart failure, stroke, and renal damage (Chockalingam, 2008). A number of factors are known to increase the risk of high BP development including obesity, sedentary lifestyle, insulin resistance, high alcohol intake, high salt intake, smoking, and aging (Carretero and Oparil, 2000; Gates et al., 2009; Green et al., 2010). The development of essential hypertension involves multiple physiological mechanisms including cardiac output, peripheral resistance, renin–angiotensin–aldosterone system, autonomic nervous system, and vasoactive substances such as endothelin, bradykinin, natriuretic peptides, and others (Beevers et al., 2001; Cain and Khalil, 2002).

The etiology of hypertension is complex and results from the interaction of multiple genetic, neuronal, hormonal, environmental factors, and aging-associated diseases (Garbers and Dubois, 1999; Oparil et al., 2003; Chockalingam, 2008). In fact, with over 50 genes implicated in BP regulation, and other risk factors contributing to the pathogenesis of hypertension, it is rarely possible to determine the etiology of the disease. However, strong evidences support the role of “vascular aging” in the development of hypertension (Green et al., 2010; Fritze et al., 2012; van den Munckhof et al., 2012). In fact, progressive aging implies endothelial dysfunction, loss of nitric oxide (NO) bioavailability, impaired vasodilation, vascular remodeling, and increased arterial stiffness. In addition, the molecular and cellular mechanisms underlying vascular alterations are common and include impaired Ca^2+^ signaling, oxidative stress, and production of pro-inflammatory cytokines and pro-fibrotic growth factors.

Regardless of its etiology, a hallmark of all cases of hypertension is an increased vascular resistance that leads to elevated BP. Resistance arteries, with an internal diameter of less than 350 μm, are key elements in the control of peripheral vascular resistance. The major drop in hydrostatic pressure in the vascular tree occurs at the level of resistance arteries. As described by Poiseuille’s law, resistance to blood flow is inversely proportional to the vessel radius to the fourth power; therefore, small variations in the lumen of resistance arteries result in significant effects on peripheral resistance with a pronounced impact on BP. Hence, peripheral resistance is typically a function of the diameter of resistance arteries which, in turn, is intricately linked to the contractility state (vasomotor tone) of vascular smooth muscle cells (VSMCs) (Bosnjak, 1993; Hill et al., 2001). Indeed, it is these VSMCs in resistance arteries and arterioles that act as the main effectors in the continuous regulation of vascular resistance. By stretching VSMCs, BP activates a myriad of signaling events that eventually produces myogenic tone, a distinguishing feature of resistance arteries and arterioles (D’Angelo et al., 1997; Davis, 2012; Mufti et al., 2015; Kroetsch et al., 2017). Furthermore, this tone represents the baseline on which various primary messengers such as neurotransmitters, endothelium-derived vasoactive molecules, local metabolites, or hormones converge and act to modulate constriction and dilatation. Many membrane channels and receptors play a pivotal role in vasotone regulation. VSMCs of resistance arteries express several plasma membrane (PM) ion channels including K^+^ channels (Taguchi et al., 1994; Sobey et al., 1998; Tajada et al., 2012), Ca^2+^ channels, Cl^−^ channels (Bulley et al., 2012; Dam et al., 2014; Heinze et al., 2014), transient receptor potential (TRP) family of ion channels, voltage-gated Ca^2+^ channels (VGCCs) (Hondeghem et al., 1986; Inoue et al., 2001; Liao et al., 2007), epithelial Na^+^/acid-sensing channel (ENaC) (Jernigan and Drummond, 2005; Drummond, 2009; Grifoni et al., 2010), and stretch-activated channel, also known as PIEZO1 (Allison, 2017). In addition to these channels, IP_3_R and ryanodine receptor (RyR), which are localized SR membrane play an important role in VSMC contractility and the development of hypertension (Long et al., 2007; Mufti et al., 2010; Lin et al., 2016).

**FIGURE 1:**
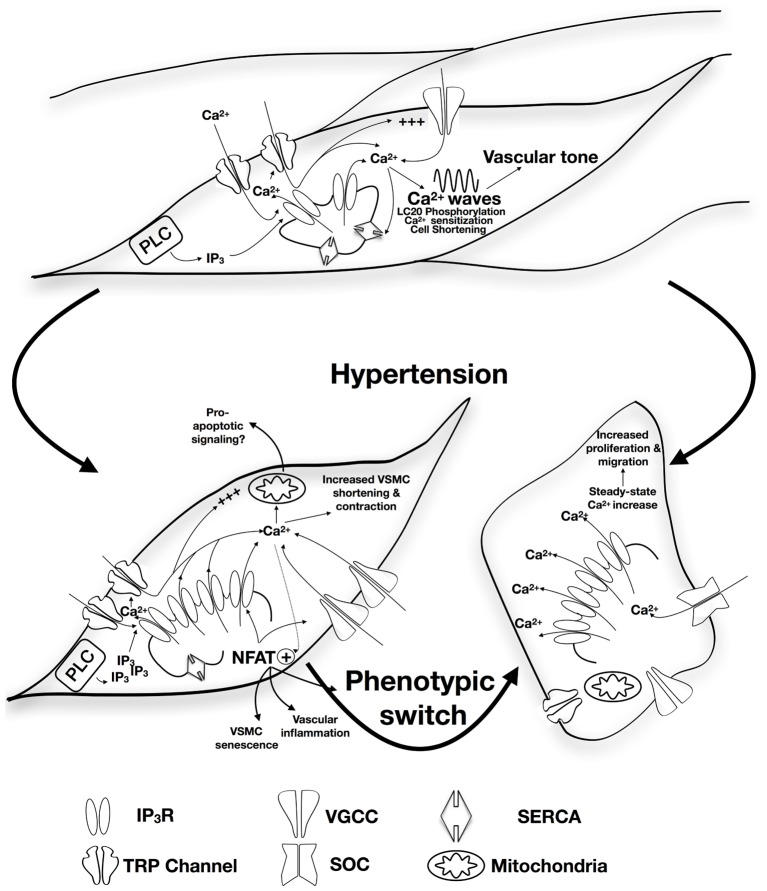
Proposed role of alteration in IP_3_R expression/function in the development of hypertension. Apart from contribution to vascular tone and VSM contractility via SR-Ca^2+^ release, calcium wave production, and induction of calcium sensitization, IP3R forms a mechanosensing complex with TRP channels that is proposed to initiate contraction in response to increased intraluminal pressure. Physiological patterns of IP_3_R-mediated Ca^2+^ release are affected by the expression of other SR membrane proteins including SERCA. Under circumstances of sustained increased blood pressure, IP_3_R expression increases together with increased sensitivity to IP_3_ and increased coupling with the TRP mechanosensing machinery resulting in increased intracellular Ca^2+^ release and increased VSM contraction. Interplay with NFAT-mediated signaling pathways might contribute pressure-induced changes in VSMC phenotype, vascular inflammation, and VSMC senescence. Increased VSMC proliferation and migration were reported to involve increased IP_3_R-mediated Ca^2+^ release with subsequent intracellular store depletion and increased store-operated Ca^2+^ entry. IP_3_R-mediated Ca^2+^ release can potentially relay apoptotic signaling to the mitochondria contributing to vascular aging.

It is important to note that with sustained hypertension, vessels undergo progressive alteration characterized by inflammatory responses, VSMC growth and migration, extracellular matrix synthesis and degradation, endothelial dysfunction that increases vascular stiffness and resistance, and decreases vascular elasticity (Shyu, 2009; Dharmashankar and Widlansky, 2010; Renna et al., 2013). Remodeled vessels heavily contribute to the pathophysiology of vascular diseases such as atherosclerosis, and are subsequently at high risk of blockage or rupture that could damage and fail the supplied organ (Renna et al., 2013). This review will highlight the role of alterations in inositol trisphosphate receptors (IP_3_R) expression/function in changes in vascular remodeling and vascular tone, and VSM contractility in response to chronic hypertension. A summary of the proposed model is presented in **Figure 1**.

## IP_3_R in VSMCs: Expression, Structure, and Localization

The IP_3_R of VSMCs plays important roles in gene expression, cellular proliferation, and migration, as well as contractility (Wilkerson et al., 2006; Xi et al., 2008; Adebiyi et al., 2010). IP_3_R is a tetramer, with each subunit encompassing an amino terminus, six transmembrane domains, and a carboxy terminal tail (Michikawa et al., 1994; Yoshikawa et al., 1996). The amino terminus contains an IP_3_-binding domain, a suppressor domain that inhibits IP_3_ binding, and a regulatory domain (Yoshikawa et al., 1999). This regulatory domain contains binding sites for Ca^2+^ and ATP as well as consensus phosphorylation sites (Michikawa et al., 1994; Patel et al., 1999; Foskett et al., 2007). Within this regulatory domain, there is also a coupling motif that is important for physical interactions between IP_3_R and transient receptor potential canonical (TRPC) channels (Tang et al., 2001; Adebiyi et al., 2010; Zhao et al., 2017). The transmembrane and carboxy terminal domains are essential for tetramerization of IP_3_Rs (Mignery and Sudhof, 1990; Sayers et al., 1997).

The IP_3_R family comprises three subtypes (IP_3_R1, IP_3_R2, and IP_3_R3) that are encoded by Itpr1, Itpr2, and Itpr3, respectively. Almost all animal cells express IP3Rs (Prole and Taylor, 2016). The human isoforms share approximately 75% amino acid homology; however, their sensitivity toward IP3 or other regulatory factors is variable, thus adding a layer of complexity for their functions (Foskett et al., 2007; Mikoshiba, 2007). This complexity is further compounded by the existence of many splice variants as well as the possibility of tetramerization (Foskett et al., 2007; Mikoshiba, 2007).

Although most cells express more than one IP3R subtype, the different subtypes exhibit some tissue-specific pattern of expression, with one subtype being expressed at a higher level than the others (Vermassen et al., 2004; Ivanova et al., 2014). Moreover, the different subtypes exhibit marked difference in their affinity for their ligand IP3 (Ivanova et al., 2014; Vervloessem et al., 2015). They also differ in their regulation by Ca^2+^ and ATP as well as their phosphorylation by various kinases (Ivanova et al., 2014; Vervloessem et al., 2015). For instance, although all three isoforms exhibit a biphasic mode of IP3-induced Ca^2+^ release, isoform-specific characteristics of this response are observed (Miyakawa et al., 1999; Mak et al., 2001; Tu et al., 2005). Likewise, ATP regulates all three isoforms but with a clear differential effect on each. For example, IP_3_R2 is 10 times more sensitive to ATP than IP_3_R3, at least in pancreatic acinar cells (Park et al., 2008). Moreover, while all three isoforms are targets for many kinases such as Akt, PKA, and MAP kinases, isoform-specific regulation by these kinases are markedly noticed. For instance, ERK1/2 can recognize three phosphorylation resides (S436, T945, and S1765) in IP_3_R1 but not in IP_3_R2 or IP_3_R3 (Bosanac et al., 2004).

Vascular smooth muscle cells expresses all three subtypes, with IP_3_R1 being the predominant one in these cells (Islam et al., 1996; Wang et al., 2001; Grayson et al., 2004; Zhou et al., 2008). Levels of these proteins are determined by a well-regulated balance between transcription and degradation. For instance, while c-Myb stimulates its expression, retinoic acid and TGF-beta inhibit expression of IP_3_R1 (Sharma et al., 1997; Deelman et al., 1998; Afroze et al., 2007). Hydrogen peroxide Jak2 kinase, Herpud1, and vasopressin regulate levels of IP_3_R1 by modulating its degradation (Sipma et al., 1998; Wallace et al., 2005; Martin-Garrido et al., 2009; Torrealba et al., 2017).

The main location of IP_3_Rs in VSMCs is the SR, both central (perinuclear), and peripheral (beneath the PM) compartments (Nixon et al., 1994; Gordienko et al., 2008; Narayanan et al., 2012). Importantly, this localization may impart a functional effect. For instance, IP_3_Rs located around the nucleus are thought to regulate Ca^2+^-dependent gene expression without affecting the global intracellular pool of Ca^2+^. On the other hand, peripherally located SR allows their IP_3_Rs to be close enough to the PM for localized signaling to membrane proteins to be efficiently elicited (Adebiyi et al., 2010; Zhao et al., 2010). It is also important to note that in addition to their role in Ca^2+^ release, IP3Rs have other significant functions. For example, upon binding, IP3 causes IP_3_R–binding protein released with IP_3_ (IRBIT) to be released from the IP3-binding site. The now released IRBIT can then modulate other targets such as transporters, channels as well as ribonucleotide reductase (Ando et al., 2003; Arnaoutov and Dasso, 2014). Moreover, IP3Rs, independent of their Ca^2+^ release ability, may also regulate others proteins such as the opening of TRPC (Zhang et al., 2001). As such, cellular distribution of IP_3_Rs and their Ca^2+^-independent roles dictate the functions of these receptors, under both physiologic and pathophysiologic conditions as will be discussed below.

## Factors Affecting Vascular Tone: Alteration in Hypertension

Under physiological conditions, individual components of the vascular system maintain a certain degree of spontaneous constriction constituting the vascular tone. This vascular property determines the dilatory capacity of the vascular bed and hence the organ, whereby a higher tone allows for a higher dilatory capacity as in the heart and skeletal muscles, and a lower tone leads to a limited dilatory capacity as in case of cerebral circulation (Klabunde, 2012). Indeed, vascular tone results from the integration of several competing stimuli that modulate the contractile state of VSMC. In isolated vessels, the myogenic response constitutes the fundamental form of vascular reactivity in response to increased intraluminal pressure (Uchida and Bohr, 1969). Extrinsic influences converge to modulate this intrinsic contractility. The overall vascular tone is set as a net outcome of the interaction of endothelial inputs activated by sheer stress (Koller et al., 1993), neuronal regulation (Fleming et al., 1987), humoral mediators (Waldemar and Paulson, 1989), tissue metabolic demand (Chovanes and Richards, 2012), and tubuloglomerular feedback (characteristic to the renal vascular bed) (Burke et al., 2014). The resultant level of constriction determines the extent of systemic vascular resistance and hence contributes to regulating BP, making the examination of alterations in vascular tone an attractive target in the study of hypertension.

Significantly, studies showed substantial alterations in vascular tone in hypertension. Whether it is a causative factor or adaptive consequence of hypertension, enhanced myogenic response was reported in humans and animal models of the disease (Henriksen et al., 1981; Sonoyama et al., 2007). Early studies on spontaneously hypertensive rats showed a reduced ability of cerebral arterioles to dilate increasing the cerebral blood flow in response to intraluminal pressure reduction (Waldemar and Paulson, 1989). Subsequent multiple reports on these animals described an enhanced myogenic constriction in response to intra-luminal pressure in different vascular beds including skeletal muscle arterioles (Falcone et al., 1993; Shibuya et al., 1998), mesenteric arteries (Matrougui et al., 2000), cerebral arterioles (Jarajapu and Knot, 2005), and renal afferent arterioles (Ren et al., 2010).

## IP_3_R-Mediated Calcium Regulation and Vascular Tone Generation: Alteration in Hypertension

Among other factors, intracellular Ca^2+^ is known to play a pivotal role in the development and maintenance of vascular myogenic tone. The increased intraluminal pressure was shown to elicit an increased intracellular Ca^2+^ in a number of vessel preparations that develop myogenic response (Schubert and Mulvany, 1999). Knot and Nelson (1998) reported a strong correlation between vessel constriction in isolated pressurized rat cerebral arteries and intraluminal pressure increase, membrane depolarization, and increased intracellular Ca^2+^. Early studies using Ca^2+^-sensitive dyes and two-dimensional electrophoresis showed that the increase in intracellular Ca^2+^ levels ([Ca^2+^]_*i*_) in these vessels was associated with an increase in myosin light chain (LC20) phosphorylation (Zou et al., 1995). Further investigation of the temporal association between increased intraluminal pressure, increased [Ca^2+^]_*i*_, and LC20 phosphorylation showed close coincidence of the three events in vessels examined in pressure myography experiments (Zou et al., 2000). Upon activation by Ca^2+^/calmodulin, the myosin light-chain kinase (MLCK) specifically phosphorylates LC20 at serine-19 (Kamm and Stull, 2001), an event that is sufficient to activate the ATPase activity of actomyosin, cross-bridge cycling, and cell shortening and contraction (Walsh et al., 1982).

Several receptors and transporters contribute to [Ca^2+^]_*i*_ dynamics in VSM, but the two primary pathways for Ca^2+^ influx are the PM L-type VGCC and the ER membrane IP_3_R (Hill et al., 2001). On the one hand, depolarization of the PM activates α_1*C*_, the pore-forming subunit of the VGCC, causing a rapid Ca^2+^ entry from extracellular space and thus leading to VSMC contraction. On the other hand, triggering of IP_3_R by IP_3_ induces Ca^2+^ release from the ER Ca^2+^ stores. The fundamental role of these two Ca^2+^ signaling pathways in the clinical management of hypertension is demonstrated by the fact that pharmacological blockers of the L-type VGCC or α-adrenergic receptors are effective in lowering BP (Oparil et al., 2003). In contractile VSMC, VGCCs are the major determinants of [Ca^2+^]_*i*_ and vascular tone. Indeed, it is mainly via through these channels that Ca^2+^ enters the cell. Nevertheless, studies also implicated RyR-mediated SR Ca^2+^ release not only as a potential contributor to the generation of myogenic tone (Mufti et al., 2010), but also via feedback regulation of VSMC depolarization through activation of large conductance Ca^2+^-dependent potassium channels (Krishnamoorthy et al., 2014).

The idea of Ca^2+^ influx through VGCC contributing to the development of myogenic response stemmed from early results demonstrating a complete loss of myogenic response following extracellular Ca^2+^ removal in a variety of vessel preparations (Schubert and Mulvany, 1999) and later corroborated by the close association between membrane potential, intracellular Ca^2+^ level, and myogenic contractility (Knot and Nelson, 1998). Voltage-associated Ca^2+^ currents were shown to occur following membrane stretching in cerebral artery (McCarron et al., 1997) and blockade of VGCC, while not affecting the depolarization produced by the increase in intraluminal pressure, inhibited the increase in vessel wall Ca^2+^ and the myogenic response (Knot and Nelson, 1998). Models proposed for this mechanotransduction process spanned the involvement of membrane integrins activating downstream Ca^2+^-sensitive and insensitive contractile pathways to a role for stretch sensitive channels (Colinas et al., 2015; Mufti et al., 2015).

Out of the several members of the VGCC family, the L-type Ca^2+^ channels received the most and earliest attention as the mediator of the extracellular Ca^2+^ influx in myogenic response. Certainly, L-type Ca^2+^ channels are broadly expressed in VSMC (Abd El-Rahman et al., 2013), and interference with Ca^2+^ influx through these channels with selective blockers was shown to preclude the myogenic response, at least partially, in many vessel preparations in early studies (McCarron et al., 1997; Knot and Nelson, 1998). On the other hand, interventions that increased L-type Ca^2+^ channel expression were associated with an increased myogenic tone (Narayanan et al., 2010). Interestingly, earlier studies of spontaneously hypertensive rats implicated increased Ca^2+^ influx via VGCC in the observed augmentation of myogenic contractility (Ren et al., 2010). It is now widely accepted that an upregulation of VGCC expression and/or function occurs in the context of hypertension (Joseph et al., 2013; Tajada et al., 2013). Several signaling proteins are implicated in this process including protein kinase C (PKC) (Joseph et al., 2013) and PI3K (Carnevale and Lembo, 2012), providing a mechanistic context for the contribution of humoral mediators such as angiotensin in increased vascular resistance.

Of interest, a model was proposed implicating a role for IP_3_R in regulating extracellular Ca^2+^ influx in VSMCs. IP_3_R activation synergistically enhanced TRP channels mediated stretch-induced depolarization (Gonzales et al., 2014). IP_3_R organizes in a signaling complex with TRPC and TRPM channels whereby stretch activates a phospholipase C isoform in addition to Ca^2+^ influx through TRPC channels. The resultant IP_3_ sensitizes IP_3_R to Ca^2+^ entering through TRPC leading to an increased SR Ca^2+^ release activating TRPM currents establishing VSMC depolarization. Significantly, the physical coupling between IP_3_R and TRP channels increased in resistance arteriole myocytes from animal models of genetic hypertension leading to an enhanced IP_3_-dependent cationic current and depolarization (Adebiyi et al., 2012).

In addition to the extracellular Ca^2+^ influx, it is well documented that Ca^2+^ release from the SR in the form of Ca^2+^ waves is involved in arterial constriction (Boittin et al., 1999; Jaggar and Nelson, 2000; Lee et al., 2005). Specifically, during the myogenic response, both the number of active cells that display Ca^2+^ waves and the frequency of these waves in a given VSMC dramatically increased upon raising the intraluminal pressure from 20 to 40 mmHg (Mufti et al., 2010). The incidence of Ca^2+^ waves at high pressure was not affected by L-type Ca^2+^ channel blockade but was rather sensitive to interference with SR Ca^2+^ release. SR Ca^2+^ depletion precluded Ca^2+^ wave production, LC20 phosphorylation, and myogenic response generation. Specifically, direct inhibition of IP_3_R was associated with impaired Ca^2+^ wave generation and interference with the myogenic tone production (Mufti et al., 2015). Similar effects of IP_3_R inhibition on micro-vessel contractility and Ca^2+^ wave production were recently observed in human tissues (Navarro-Dorado et al., 2014). Importantly, the expression of several SR and PM-associated Ca^2+^ handling proteins, including IP_3_R, sarco/endoplasmic reticulum Ca^2+^ ATPase (SERCA), and Na^+^/Ca^2+^ exchanger, was upregulated in different hypertensive animal models. Together with an increased SR Ca^2+^ release, the upregulation of these proteins leads to enhanced basal and evoked vascular constriction (Linde et al., 2012; Abou-Saleh et al., 2013). Specifically, IP_3_R expression was shown to be higher in VSMC from spontaneously hypertensive rats compared to non-hypertensive controls (Bernier and Guillemette, 1993). Moreover, in rat models of genetic hypertension, both IP_3_ production and IP_3_/IP_3_R-binding affinity are increased (Wu and de Champlain, 1996) together with an increased global [Ca^2+^]_*i*_ (Jarajapu and Knot, 2005).

Yet, Osol et al. (2002) showed that increased force production in a myogenic vessel preparation in the pressure range that is associated with myogenic contractility (60–140 mmHg) was not associated with appreciable increases in membrane depolarization or intracellular Ca^2+^ concentration. As well, a number of early studies raised the possibility of the involvement of Ca^2+^-independent force generation mechanisms in myogenic contractility. Calcium–tone relationships were shown to be fivefold more sensitive during pressure-induced activation (VanBavel et al., 1998), and myogenic contractility persisted in situations with reduced extracellular Ca^2+^ levels (McCarron et al., 1997) or where membrane potential was clamped in a depolarized state with high extracellular potassium precluding further Ca^2+^ entry (McCarron et al., 1997; Lagaud et al., 2002). Multiple lines of evidence implicated signaling pathways involving activation of G proteins, PKC, and Rho-associated protein kinase (ROK) in the generation of Ca^2+^-independent contraction within the context of the vascular tone development (McCarron et al., 1997; VanBavel et al., 2001; Wesselman et al., 2001; Lagaud et al., 2002). Interestingly, a growing body of evidence suggests that enhanced Ca^2+^ sensitization contributes to augmented vascular tone in models of hypertension (Uehata et al., 1997; Jarajapu and Knot, 2005; Zicha et al., 2014; Behuliak et al., 2017). However, despite the direct observations that a sustained arteriolar constriction could be obtained via enhanced actin cytoskeleton reorganization triggered by a seemingly Ca^2+^-independent signaling pathways (ROK- and PKC-mediated pathways) (Moreno-Domínguez et al., 2013, 2014; Colinas et al., 2015; El-Yazbi et al., 2015). These studies clearly demonstrated the obligate dependence of the generation of arteriolar vascular tone on Ca^2+^. Not only interferences with extracellular Ca^2+^ levels affected the myogenic response and the mechanisms of all force generation including the Ca^2+^-independent pathways; specific inhibition of IP_3_R precluded a pressure-dependent increase in Ca^2+^ sensitization (Mufti et al., 2015).

## Alterations in IP_3_R Expression/Activity Associated With Vascular Remodeling

Apart from mechanisms contributing to vascular tone through regulation of the contractile machinery, an additional interesting factor is the alteration in structural properties of the vessel wall, referred to as vascular remodeling, a phenomenon strongly associated with age (Baek and Kim, 2011). Initially, vascular remodeling constituted an adaptive response of VSMC to hemodynamic changes that can be sensed by vascular cells, both endothelial and SMCs and translated into structural alteration within the vessel wall. On the long run, however, these adaptations lead to increased media thickness, reduced luminal diameter, and extracellular matrix reorganization (Mulvany et al., 1996; Touyz, 2005; Lemarie et al., 2010; Rizzoni and Agabiti-Rosei, 2012). Furthermore, vascular injury induced by disruption of atheromatous plaque or balloon angioplasty triggers a reparative response that includes inflammation, migration and proliferation of VSMC, and intimal hyperplasia. Ultimately, due to changes in vessel architecture and geometry, this leads to a negative constrictive remodeling of the arterial wall (Gibbons and Dzau, 1994; Faxon et al., 1997).

It is now accepted that structural remodeling in resistance arteries is closely related to the development of hypertension (Lemarie et al., 2010; Rizzoni and Agabiti-Rosei, 2012). In this perspective, smooth muscle cells display a significant degree of phenotypic plasticity and, unlike most other differentiated cells, can change their phenotype even at the differentiated state (Yoshida and Owens, 2005; Matchkov et al., 2012). This involves a phenotypic switch from a contractile to a proliferative, migrating, and or/synthetic phenotype and is associated with gene regulation and alteration of cytoplasmic Ca^2+^ signaling machinery (House et al., 2008; Matchkov et al., 2012). While vascular remodeling in aging has been partially investigated (Wang et al., 2005, 2006, 2007), the molecular mechanisms involved in the remodeling of Ca^2+^ signaling pathways observed in hypertension is still poorly understood.

In VSMCs, resting [Ca^2+^]_*i*_ is slightly higher than in other cells, allowing the vessel to be in a constant state of partial contraction. In the synthetic phenotype, however, this turns to be less important or even voltage-independent. In contrast to the role proposed for VGCC and IP_3_R in VSM contraction, it has been suggested that regulation of [Ca^2+^]_*i*_ in synthetic VSMC occurs via alternative pathways including store-operated channels (SOCs) and receptor-operated channels (ROCs) (Berra-Romani et al., 2008; Baryshnikov et al., 2009). SOCs are activated by depletion of internal Ca^2+^ stores mainly through IP_3_-mediated Ca^2+^ release (Trebak, 2012), whereas ROCs activation involves different components of the PLC signaling cascade including IP_3_ (House et al., 2008). It is beyond the scope of this review to discuss these two pathways in more detail. Of note, however, the expression level of all three IP_3_R isoforms increase during VSMC switch from contractile to synthetic phenotype (Berra-Romani et al., 2008). Additionally, IP_3_R-mediated Ca^2+^ release increases in proliferating VSMC offering a possible explanation for the observed increased in SOC Ca^2+^ entry (Moses et al., 2001; Wilkerson et al., 2006). Selective inhibition of IP_3_R not only reduced VSMC proliferation (Wang et al., 2001; Wilkerson et al., 2006) but also inhibited *in vitro* pressure-induced increase in VSMC migration (Tada et al., 2008). Evidence in synthetic human VSMCs point to an altered mode of Ca^2+^ release via IP_3_R (Bobe et al., 2011). IP_3_-mediated release in these cells occurs in a steady state followed by store-operated calcium entry. This pattern was restored to the oscillatory Ca^2+^ release pattern characteristic to contractile VSMCs upon upregulation of SERCA pump expression. This switch reduced nuclear factor of activated T cells (NFAT) signaling. In the context of hypertension, we have previously shown that L-type Ca^2+^ channels and IP_3_R are specifically and concomitantly upregulated in an angiotensin-induced hypertension model through a NFAT-dependent pathway (Abou-Saleh et al., 2013). Functionally, this was associated with enhancement and sensitization of IP_3_-dependent Ca^2+^ release, thereby resulting in higher basal Ca^2+^ levels and increased VSM contraction. In addition to hypertension, upregulated NFAT signaling in the vasculature was implicated in a number of age-related disorders including post-injury restenosis (Bonnet et al., 2009), vascular inflammation, and aggravation of atherosclerosis in diabetes (Nilsson-Berglund et al., 2010; Zetterqvist et al., 2014), as well as vascular smooth muscle senescence (Min et al., 2009). This latter observation taken together with the evidence regarding the association of increased IP_3_R expression/activity, NFAT signaling, and VSMC phenotypic switch may add novel insights into the role of IP_3_R in VSMC molecular remodeling as a part of the aging process. Specifically, studies in several cell types demonstrated that different IP_3_R isoforms occur in close proximity to the mitochondria and transmit pro-apoptotic Ca^2+^ signals (Simpson et al., 1998; Szalai et al., 1999; Mendes et al., 2005). Yet, it is worth mentioning that the role of IP_3_R in aging is far from being clear. Whereas IP_3_ content was shown to increase in rat brain (Igwe and Ning, 1993), IP_3_R expression and IP_3_ binding were shown to be decreased (Igwe and Filla, 1997).

In addition to NFAT, other Ca^2+^ sensitive transcription factors such as serum response factor (SRF), c-response element binding (CREB) seem to play an important role in switching VSMC from a contractile to a synthetic phenotype (Matchkov et al., 2012). Future studies on the role of IP_3_R in this process need to be conducted in IP_3_R-deficient mice. In this regard, the role of IP_3_R in VSMC contractility *in vivo* was recently highlighted in a conditional triple knockout mouse, where the agonist-mediated vascular constriction was attenuated together with a lack of development of hypertension in response to chronic angiotensin infusion (Lin et al., 2016). However, the effect of the conditional knockout on VSMC phenotypic switch in response to hypertension has not been addressed so far.

## Conclusions and Future Perspectives

Hypertension is a complex disease that arises from the interaction of multiple genetic, environmental, nutritional, hormonal, and age-related pathological conditions. The etiology of “essential hypertension,” which accounts for more than 90% of clinical hypertension, comprises an increased vascular resistance and is associated with structural alterations in the wall of resistance arteries. Modulation of [Ca^2+^]_*i*_ in VSMC allows small arteries and arterioles to establish vasomotor tone and regulate blood flow, and determine peripheral vascular resistance and BP. These changes require a phenotypic switch of VSMC from a contractile quiescent to a versatile proliferative phenotype, a phenomenon widely observed in age-associated vascular remodeling. As described above, IP_3_R activity was shown to be essential in almost every cellular mechanism involved in setting vascular tone level. Additionally, modulation of IP_3_-dependent Ca^2+^ signaling may represent an essential stimulus for VSMC shift from quiescent to the proliferative state. KT-362 was an investigational drug targeting IP_3_R-mediated Ca^2+^ release that showed a clinically relevant antihypertensive action (Hester and Shibata, 1990). However, clinical trials were discontinued at phase II. In addition to known targets for antihypertensive therapy, novel interventions within the PLC–IP_3_R pathway constitute attractive therapeutic targets for future research given their ubiquitous involvement in cellular processes leading to hypertension.

## Author Contributions

AE and HA-S designed and wrote the first draft of the manuscript. AE-Y designed the graphical abstract and reviewed the manuscript. FZ, AA, AO, and MR reviewed the manuscript. HZ, GP, and HA-S proofread and revised the manuscript.

## Conflict of Interest Statement

The authors declare that the research was conducted in the absence of any commercial or financial relationships that could be construed as a potential conflict of interest.
